# Supplementary material for: Employment Industry and Occupational Class in Relation to Serious Psychological Distress in the United States
Source: Int J Environ Res Public Health. 2022 Jul 8;19(14):8376. doi: 10.3390/ijerph19148376 (PMC9320061; doi:10.3390/ijerph19148376)
Supplement: Supplementary file 1 [file ijerph-19-08376-s001.zip › ijerph-1777054-supplementary.pdf]

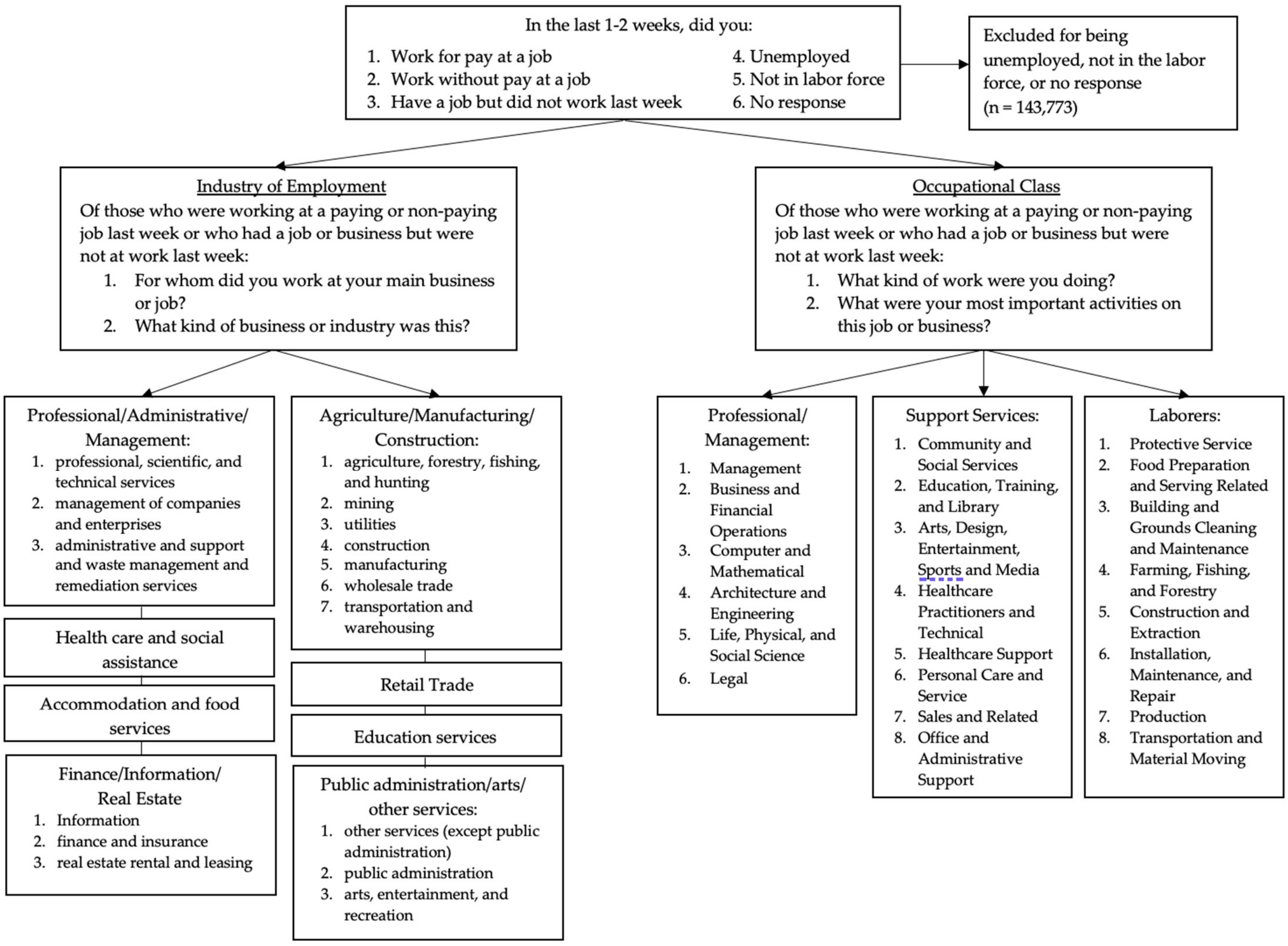

Figure S1. Breakdown of Occupation Data into Occupational Classes and Industries of Employment.

Table S1. Age-Standardized [a] Occupational Class and Industry of Employment Characteristics among U.S. Adults Overall, by Race/Ethnicity, and stratified by Serious Psychological Distress (SPD), National Health Interview Survey, 2004-2018, (N=245,038).

	Race/Ethnicity												Total (N=245,038)		
	Asian (N=13,488)			NH-Black (N=32,164)			Hispanic/Latinx (N=43,268)			NH-White (N=156,118)					
	All N=13,488	No SPD N=13,329	With SPD N=159	All N=32,164	No SPD N=31,514	With SPD N=650	All N=43,268	No SPD N=42,269	With SPD N=999	All N=156,118	No SPD N=153,320	With SPD N=2,798	All N=245,038	No SPD N=240,432	With SPD N=4,606
SPD [b] (% yes)	1.0			1.6			2.1			1.4			1.5		
**Industry of Employment**															
Professional/Administrative/ Management [c] (%)	14.8	14.8	17.2	9.9	9.9	10.6	10.9	10.9	12.2	11.7	11.7	10.8	11.6	11.6	11.3
Agriculture/Manufacturing/ Construction [d] (%)	23.6	23.7	13.2	22.3	22.3	20.5	32.7	32.9	19.9	27.1	27.2	23.4	27.2	27.3	22.2
Retail Trade (%)	8.9	8.8	15.0	8.4	8.4	13.1	9.6	9.4	14.9	10.7	10.7	14.5	10.3	10.3	14.3
Finance/Information/Real Estate [e] (%)	9.6	9.6	10.2	7.1	7.1	5.1	6.4	6.5	4.6	9.9	10.0	6.1	9.3	9.3	6.0
Educational services (%)	8.3	8.3	6.4	10.5	10.5	7.4	7.0	7.0	7.5	10.7	10.8	8.7	10.1	10.1	8.2
Health care/Social assistance (%)	16.1	16.0	20.2	21.0	20.9	28.1	12.3	12.2	16.9	12.8	12.8	15.5	13.6	13.5	17.2
Accommodation/Food services (%)	6.9	6.9	3.9	5.4	5.3	7.1	8.3	8.2	11.1	4.2	4.1	7.7	5.0	4.9	8.0
Public administration/Arts/Other services (%)	11.8	11.8	14.1	15.4	15.5	8.1	12.8	12.8	12.9	12.8	12.8	13.3	13.0	13.0	12.8
**Occupational Class**															
Professional/Management (%)	35.2	35.4	24.1	14.5	14.6	8.0	11.6	11.7	8.5	24.8	25.0	13.9	22.2	22.3	12.5
Support services (%)	44.0	43.9	52.4	49.2	49.2	52.6	34.8	34.7	40.6	46.3	46.3	49.3	44.8	44.7	48.1
Laborers (%)	20.7	20.7	23.5	36.2	36.2	39.4	53.6	53.7	50.9	28.8	28.7	36.8	33.1	32.9	39.4
**Professional/Administrative/ Management**															
Professional/Management (%)	77.1	77.1	71.5	29.1	29.4	14.7	22.4	22.5	19.9	54.2	54.6	30.6	48.8	49.1	27.7
Support services (%)	15.5	15.6	7.9	30.5	30.3	39.2	18.7	18.6	21.7	27.3	27.2	34.6	25.4	25.3	31.8
Laborers (%)	7.4	7.3	20.7	40.4	40.3	46.1	58.9	58.9	58.4	18.5	18.2	34.8	25.8	25.5	40.5
**Agriculture/Manufacturing/Construction**															
Professional/Management (%)	36.4	36.4	40.7	10.7	10.7	10.0	9.0	9.1	7.6	25.8	25.9	14.5	21.7	21.8	13.3
Support services (%)	20.2	20.3	6.0	19.5	19.4	22.7	12.1	12.1	15.4	19.9	19.8	20.9	18.4	18.3	19.5
Laborers (%)	43.4	43.3	53.4	69.8	69.9	67.3	78.9	78.9	77.0	54.4	54.2	64.6	60.0	59.9	67.2
**Retail Trade**															
Professional/Management (%)	10.5	10.7	0.0	4.5	4.5	2.4	4.0	3.9	7.2	7.2	7.3	4.3	6.6	6.6	4.4
Support services (%)	77.1	77.0	80.6	75.5	75.4	80.7	72.4	72.6	61.4	75.5	75.4	77.9	75.1	75.1	75.7
Laborers (%)	12.4	12.3	19.4	20.0	20.1	16.9	23.7	23.5	31.4	17.3	17.3	17.8	18.3	18.3	19.9
**Finance/Information/Real Estate**															

Professional/Management (%)	54.4	54.4	49.8	35.8	35.8	36	31.4	31.7	17.1	42.8	42.9	32.9	41.6	41.7	30.9
Support services (%)	41.4	41.4	50.2	50.2	50.2	50.2	49.5	49.3	61.9	49.2	49.1	57.3	48.9	48.8	57.3
Laborers (%)	4.2	4.2	0.0	14.0	14.0	13.8	19.0	19.0	21.0	8.0	7.9	9.8	9.5	9.5	11.8
**Educational services**															
Professional/Management (%)	20.1	20.3	4.2	11.6	11.7	1.1	10.6	10.5	10.8	12.7	12.8	11.3	12.7	12.8	9.8
Support services (%)	71.1	71.0	84.3	66.8	66.9	61.3	70.3	70.2	78.6	77.2	77.2	70.1	75.1	75.2	71.0
Laborers (%)	8.8	8.7	11.5	21.6	21.4	37.6	19.1	19.3	10.6	10.1	10.0	18.6	12.1	12.0	19.2
**Health care/Social assistance**															
Professional/Management (%)	12.7	12.7	7.2	7.3	7.4	1.9	7.4	7.6	2.4	12.3	12.4	5.1	10.9	11.0	4.2
Support services (%)	83.3	83.2	87.2	82.1	82.0	83.5	82.6	82.4	90.5	82	81.9	84.4	82.1	82.1	85.5
Laborers (%)	4.1	4.1	5.6	10.6	10.6	14.6	9.9	10.0	7.2	5.8	5.7	10.5	7.0	6.9	10.3
**Accommodation/Food services**															
Professional/Management (%)	19.1	18.9	40.9	10.3	10.5	4.7	8.5	8.6	4.5	17.5	17.8	10.2	14.5	14.6	8.8
Support services (%)	15.4	15.5	0.0	17.8	17.8	18.1	12.0	12.1	10.4	12.0	11.9	14	12.9	12.9	13.4
Laborers (%)	65.5	65.6	59.1	71.9	71.8	77.3	79.5	79.3	85.1	70.5	70.3	75.8	72.6	72.5	77.8
**Public administration/ Arts/Other services**															
Professional/Management (%)	25.4	25.5	17.7	20.1	20.2	12.1	11.0	11.1	6.3	22.9	23.0	12.7	20.9	21.0	11.6
Support services (%)	54.8	54.8	56.8	46.4	46.3	52.4	36.4	36.6	30.7	45.9	45.8	57.3	45.0	44.9	51.7
Laborers (%)	19.8	19.7	25.5	33.5	33.5	35.4	52.6	52.4	62.9	31.2	31.2	29.9	34.1	34.0	36.6

Abbreviations: kg=kilograms; m=meters; NH=non-Hispanic; PA=physical activity; SE=standard error; SPD=serious psychological distress

[a] All estimates are weighted for the survey's complex sampling design. All estimates are age-standardized to the U.S. 2010 population. Percentages may not sum to 100 due to missing values or rounding.

[b] Kessler-6 psychological distress scale score ≥ 13

[c] Includes the following NAICS (NAICS Association, LLC, Rockaway, New Jersey) industry categories: information; finance and insurance; and real estate rental and leasing.

[d] Includes the following NAICS industry categories: agriculture, forestry, fishing, and hunting; mining; utilities; construction; manufacturing; wholesale trade; and transportation and warehousing.

[e] Includes the following NAICS industry categories: professional, scientific, and technical services; management of companies and enterprises; and administrative support and waste management and remediation services industries.

[f] Includes the following NAICS industry categories: public administration; arts, entertainment, and recreation; and other services (except public administration).

Table S2. Adjusted Prevalence Ratios (95% Confidence Intervals) for the Association Between Occupational Class (compared to Professional/Management positions) and Industry of Employment (compared to the Professional/Administrative/Management Industry) and Serious Psychological Distress, Stratified by Race/Ethnicity and Gender, National Health Interview Survey, 2004-2018 (N=245,038).

	**Race/Ethnicity**							
	**Asian**		**NH-Black**		**Hispanic/Latinx**		**NH-White**	
**Gender**	Women (N=6,413)	Men (N=7,075)	Women (N=18,955)	Men (N=13,209)	Women (N=20,308)	Men (N=22,960)	Women (N=77,958)	Men (N=78,160)
**Occupational Class** [a]								
Support services	1.38 [0.57 - 3.34]	1.61 [0.63 - 4.14]	1.32 [0.81 - 2.15]	1.14 [0.62 - 2.10]	1.05 [0.71 - 1.57]	1.72 [0.98 - 3.03]	**1.25 [1.02 - 1.53]**	1.15 [0.89 - 1.50]
Laborers	1.46 [0.58 - 3.64]	1.15 [0.41 - 3.2]	1.38 [0.83 - 2.31]	1.01 [0.55 - 1.86]	1.18 [0.76 - 1.82]	1.28 [0.71 - 2.33]	**1.45 [1.14 - 1.85]**	1.02 [0.80 - 1.31]
**Industry of Employment** [b]								
Manufacturing/Construction	1.19 [0.31 - 4.58]	1.14 [0.38 - 3.44]	0.83 [0.48 - 1.43]	0.76 [0.38 - 1.53]	0.81 [0.54 - 1.21]	0.77 [0.47 - 1.27]	0.85 [0.65 - 1.11]	0.80 [0.63 - 1.03]
Retail Trade	1.94 [0.63 - 6.01]	**4.10 [1.36 - 12.4]**	0.65 [0.37 - 1.16]	1.38 [0.60 - 3.17]	0.85 [0.54 - 1.35]	1.09 [0.57 - 2.09]	0.99 [0.77 - 1.28]	0.78 [0.56 - 1.08]
Finance/Information/Real Estate	1.01 [0.35 - 2.95]	0.81 [0.18 - 3.53]	0.55 [0.29 - 1.03]	0.31 [0.09 - 1.04]	1.00 [0.56 - 1.79]	0.80 [0.36 - 1.81]	**0.71 [0.52 - 0.97]**	0.68 [0.46 - 1.01]
Educational Services	0.85 [0.29 - 2.46]	3.10 [0.63 - 15.3]	**0.56 [0.32 - 1.00]**	0.93 [0.30 - 2.84]	0.69 [0.40 - 1.20]	1.06 [0.41 - 2.78]	**0.66 [0.49 - 0.89]**	1.20 [0.82 - 1.76]
Healthcare/Social Assistance	1.36 [0.48 - 3.85]	2.79 [0.64 - 12.3]	**0.57 [0.35 - 0.92]**	1.31 [0.57 - 3.01]	0.93 [0.63 - 1.36]	**2.15 [1.04 - 4.44]**	0.89 [0.71 - 1.13]	0.92 [0.60 - 1.41]
Accommodation/Food services	0.30 [0.09 - 1.00]	0.48 [0.11 - 2.07]	0.62 [0.33 - 1.17]	1.02 [0.42 - 2.50]	1.16 [0.77 - 1.76]	0.60 [0.31 - 1.14]	1.15 [0.88 - 1.52]	0.79 [0.54 - 1.14]
Public Administration/Arts/Other services	1.06 [0.37 - 3.01]	2.20 [0.68 - 7.19]	**0.39 [0.21 - 0.73]**	0.86 [0.42 - 1.74]	0.72 [0.46 - 1.11]	0.90 [0.41 - 1.96]	0.90 [0.70 - 1.16]	**0.69 [0.51 - 0.94]**
Professional/Administrative/Management								
Support services	NE	NE	1.11 [0.30 - 4.07]	NE	0.89 [0.37 - 2.15]	1.68 [0.46 - 6.10]	1.16 [0.68 - 1.97]	**2.04 [1.22 - 3.40]**
Laborers	NE	NE	0.93 [0.20 - 4.28]	NE	1.02 [0.40 - 2.57]	1.00 [0.22 - 4.62]	1.26 [0.70 - 2.26]	**1.91 [1.18 - 3.11]**
Manufacturing/Construction								
Support services	NE	NE	1.45 [0.26 - 8.23]	0.62 [0.18 - 2.11]	0.88 [0.31 - 2.53]	1.24 [0.48 - 3.23]	1.31 [0.80 - 2.14]	1.08 [0.57 - 2.06]
Laborers	NE	NE	1.42 [0.23 - 8.93]	0.79 [0.27 - 2.34]	0.80 [0.38 - 1.69]	0.99 [0.43 - 2.32]	1.56 [0.98 - 2.49]	1.06 [0.68 - 1.65]
Retail Trade								
Support services	NE	NE	3.78 [0.43 - 33.5]	0.46 [0.05 - 4.68]	**0.22 [0.06 - 0.78]**	NE	2.16 [0.92 - 5.03]	0.76 [0.29 - 1.99]
Laborers	NE	NE	3.26 [0.31 - 34.4]	0.39 [0.04 - 3.98]	0.52 [0.14 - 1.92]	NE	1.97 [0.7 - 5.52]	0.77 [0.26 - 2.29]

Finance/Information/Real Estate								
Support services	NE	NE	0.52<br>[0.17 - 1.54]	NE	1.49<br>[0.51 - 4.36]	NE	0.93<br>[0.54 - 1.63]	0.66<br>[0.30 - 1.43]
Laborers	NE	NE	0.46<br>[0.06 - 3.32]	NE	0.58<br>[0.08 - 4.12]	NE	0.40<br>[0.10 - 1.57]	1.09<br>[0.46 - 2.56]
Educational Services								
Support services	NE	NE	NE	NE	0.38<br>[0.09 - 1.60]	0.50<br>[0.07 - 3.43]	0.93<br>[0.42 - 2.04]	1.54<br>[0.61 - 3.86]
Laborers	NE	NE	NE	NE	0.14<br>[0.02 - 1.17]	0.26<br>[0.03 - 2.42]	1.36<br>[0.50 - 3.73]	0.59<br>[0.20 - 1.79]
Health Care/Social Assistance								
Support services	NE	NE	1.62<br>[0.40 - 6.62]	NE	2.13<br>[0.64 - 7.12]	6.26<br>[0.51 - 77.5]	2.22<br>[0.87 - 5.70]	0.76<br>[0.30 - 1.94]
Laborers	NE	NE	1.27<br>[0.26 - 6.27]	NE	1.38<br>[0.31 - 6.15]	1.95<br>[0.10 - 39.6]	2.49<br>[0.90 - 6.93]	0.31<br>[0.08 - 1.16]
Accommodation/Food Services								
Support services	NE	NE	1.88<br>[0.36 - 9.82]	NE	0.89<br>[0.26 - 3.10]	NE	2.06<br>[0.91 - 4.64]	0.92<br>[0.30 - 2.81]
Laborers	NE	NE	3.30<br>[0.70 - 15.5]	NE	1.23<br>[0.44 - 3.43]	NE	**2.15**<br>**[1.00 - 4.61]**	0.53<br>[0.19 - 1.44]
Public Administration/Arts/Other Services								
Support services	NE	0.49<br>[0.07 - 3.36]	3.00<br>[0.76 - 11.8]	0.64<br>[0.20 - 2.07]	1.31<br>[0.26 - 6.6]	0.41<br>[0.05 - 3.38]	1.24<br>[0.74 - 2.09]	1.34<br>[0.60 - 2.98]
Laborers	NE	0.53<br>[0.04 - 6.65]	1.04<br>[0.25 - 4.37]	0.72<br>[0.17 - 3.05]	1.89<br>[0.35 - 10.3]	1.30<br>[0.17 - 10.1]	1.10<br>[0.56 - 2.16]	0.81<br>[0.35 - 1.88]

Abbreviations: NH=non-Hispanic; NE=not estimable

[a] Reference group: professional/management positions

[b] Reference group: professional/administrative/management industry

All models adjusted for age (18-30, 31-49, ≥50 years), gender (women, men), educational attainment (<high school, high school graduate, some college, ≥college), annual household income (<$35,000, $35,000-$74,999, $75,000+), region of residence (Northeast, Midwest, South, West), marital/co-habiting status (married/living with partner or cohabitating, divorced/widowed/separated, single/no live-in partner), health status (excellent/very good, good, fair/poor), alcohol consumption (never, former, current), and "ideal" cardiovascular health (never smoking/quit >12 months prior to interview, BMI 18.5-<25 kg/m, meeting physical activity guidelines, and no prior diagnosis of dyslipidemia, hypertension, or diabetes/prediabetes).

All estimates are weighted for the survey's complex sampling design. Boldface indicates statistically significant results at the 0.05 level

Overall models adjusted for race/ethnicity (NH-White, NH-Black, Hispanic/Latinx, and Asian).

Table S3. Adjusted Prevalence Ratios (95% Confidence Intervals) for the Association Between Occupational Class (compared to Professional/Management positions) and Industry of Employment (compared to the Professional/Administrative/Management Industry) and Serious Psychological Distress, Stratified by Race/Ethnicity and Age, National Health Interview Survey, 2004-2018, (N=245,038).

	**Race/Ethnicity**							
	**Asian**		**NH-Black**		**Hispanic/Latinx**		**NH-White**	
**Age**	<50 years (N=9,901)	≥50 years (N=3,587)	<50 years (N=22,463)	≥50 years (N=9,701)	<50 years (N=34,440)	≥50 years (N=8,828)	<50 years (N=97,407)	≥50 years (N=58,711)
**Occupational Class [a]**								
Support services	1.21 [0.57 - 2.53]	5.09 [0.95 - 27.3]	1.16 [0.74 - 1.79]	1.49 [0.68 - 3.25]	1.08 [0.77 - 1.53]	2.21 [0.87 - 5.61]	**1.24 [1.02 - 1.50]**	0.99 [0.75 - 1.31]
Laborers	1.13 [0.5 - 2.52]	3.63 [0.77 - 17.1]	1.10 [0.69 - 1.75]	1.51 [0.65 - 3.51]	1.03 [0.71 - 1.50]	**2.70 [1.03 - 7.06]**	**1.24 [1.00 - 1.55]**	1.07 [0.82 - 1.40]
**Industry of Employment [b]**								
Manufacturing/Construction	1.59 [0.69 - 3.68]	0.39 [0.09 - 1.63]	0.73 [0.44 - 1.2]	0.75 [0.32 - 1.73]	0.91 [0.64 - 1.31]	**0.44 [0.22 - 0.87]**	0.83 [0.66 - 1.05]	0.85 [0.61 - 1.17]
Retail Trade	**3.07 [1.31 - 7.16]**	1.92 [0.43 - 8.63]	0.85 [0.49 - 1.47]	1.09 [0.40 – 3.00]	0.96 [0.63 - 1.45]	0.76 [0.32 - 1.82]	0.94 [0.75 - 1.19]	0.78 [0.54 - 1.13]
Finance/Information/Real Estate	1.31 [0.54 - 3.14]	0.39 [0.06 - 2.59]	**0.49 [0.26 - 0.93]**	0.64 [0.21 - 1.92]	1.17 [0.69 - 1.98]	**0.33 [0.13 - 0.79]**	**0.65 [0.48 - 0.87]**	0.84 [0.54 - 1.31]
Educational Services	1.77 [0.57 - 5.43]	0.95 [0.2 - 4.55]	0.83 [0.45 - 1.54]	**0.34 [0.13 - 0.93]**	0.82 [0.47 - 1.43]	0.54 [0.23 - 1.29]	**0.73 [0.54 - 0.97]**	0.91 [0.60 - 1.39]
Healthcare/Social Assistance	1.45 [0.63 - 3.37]	2.12 [0.35 - 12.8]	0.70 [0.43 - 1.15]	0.75 [0.30 - 1.85]	1.26 [0.87 - 1.83]	0.57 [0.29 - 1.11]	**0.78 [0.61 - 0.98]**	1.11 [0.78 - 1.59]
Accommodation/Food services	0.36 [0.13 – 1.00]	0.32 [0.04 - 2.31]	0.82 [0.46 - 1.44]	0.62 [0.20 - 1.96]	1.11 [0.76 - 1.61]	0.49 [0.16 - 1.47]	1.05 [0.82 - 1.36]	0.77 [0.47 - 1.25]
Public Administration/Arts/Other services	1.73 [0.78 - 3.85]	0.80 [0.18 - 3.62]	0.65 [0.39 - 1.09]	**0.32 [0.12 - 0.84]**	0.93 [0.59 - 1.47]	**0.42 [0.19 - 0.93]**	0.84 [0.66 - 1.07]	0.72 [0.50 - 1.05]
**Professional/Administrative/Management**								
Support services	NE	NE	1.36 [0.46 - 4.04]	2.74 * [0.36 - 20.7]	0.94 [0.41 - 2.15]	2.11 * [0.32 - 13.8]	**1.89 [1.24 - 2.89]**	0.79 [0.36 - 1.71]
Laborers	NE	NE	1.06 [0.35 - 3.20]	3.09 * [0.43 - 22.2]	1.12 [0.38 - 3.26]	1.03 * [0.17 - 6.35]	1.55 [0.96 - 2.48]	1.43 [0.70 - 2.93]
**Manufacturing/Construction**								
Support services	0.22 [0.04 - 1.33]	NE	1.03 [0.33 - 3.18]	1.09 * [0.21 - 5.61]	0.76 [0.33 - 1.78]	8.06 [0.95 - 68.4]	1.30 [0.75 - 2.25]	0.84 [0.50 - 1.43]
Laborers	0.58 [0.14 - 2.51]	NE	1.18 [0.34 - 4.12]	0.78 [0.18 - 3.42]	0.70 [0.37 - 1.34]	4.76 [0.64 - 35.5]	1.33 [0.85 - 2.10]	0.98 [0.63 - 1.54]
**Retail Trade**								
Support services	NE	NE	1.25 [0.15 - 10.0]	1.10 * [0.04 - 27.5]	1.47 [0.17 - 12.8]	0.18 [0.03 - 1.17]	1.87 [0.89 - 3.91]	0.60 [0.19 - 1.88]
Laborers	NE	NE	1.17 [0.12 - 10.9]	0.84 * [0.02 - 41.8]	1.72 [0.19 - 15.5]	1.45 [0.16 - 13.4]	1.80 [0.77 - 4.22]	0.63 [0.17 - 2.28]
**Finance/Information/Real Estate**								
Support services	NE	NE	0.55 * [0.16 - 1.84]	NE	1.80 [0.73 - 4.45]	NE	1.08 [0.65 - 1.81]	0.60 [0.28 - 1.29]

Laborers	NE	NE	0.73 * [0.13 - 4.26]	NE	1.63 [0.54 - 4.96]	NE	0.77 [0.29 - 2.07]	0.77 [0.29 - 2.01]
**Educational Services**								
Support services	NE	NE	7.34 [0.85 - 63.1]	NE	0.37 [0.09 - 1.58]	NE	0.91 [0.44 - 1.85]	1.88 [0.62 - 5.72]
Laborers	NE	NE	6.67 [0.60 - 73.7]	NE	**0.11 [0.02 - 0.66]**	NE	0.58 [0.24 - 1.38]	3.19 [0.97 - 10.5]
**Health Care/Social Assistance**								
Support services	0.50 [0.05 - 5.30]	NE	1.92 [0.46 - 8.13]	NE	2.58 [0.70 - 9.46]	4.28 * [0.51 - 36.1]	1.48 [0.61 - 3.57]	1.59 [0.72 - 3.49]
Laborers	2.38 [0.09 - 61.1]	NE	1.60 [0.35 - 7.37]	NE	1.35 [0.26 - 6.91]	1.98 * [0.21 - 18.9]	1.13 [0.40 - 3.18]	1.70 [0.62 - 4.64]
**Accommodation/Food Services**								
Support services	NE	NE	2.16 [0.43 - 10.8]	NE	0.70 [0.22 - 2.22]	NE	1.37 [0.71 - 2.63]	2.63 * [0.52 - 13.3]
Laborers	NE	NE	2.68 [0.65 - 11.1]	NE	1.16 [0.49 - 2.72]	NE	1.23 [0.69 - 2.21]	3.31 * [0.76 - 14.4]
**Public Administration/Arts/Other Services**								
Support services	1.41 * [0.45 - 4.43]	0.07 * [0.00 - 5.18]	1.14 [0.45 - 2.88]	3.75 * [0.33 - 42.2]	1.08 [0.28 - 4.14]	NE	1.34 [0.78 - 2.30]	0.96 [0.44 - 2.07]
Laborers	1.51 * [0.30 - 7.73]	4.52 * [0.49 - 41.5]	0.60 [0.18 - 2.01]	5.48 * [0.45 - 66.3]	1.27 [0.31 - 5.21]	NE	1.06 [0.55 - 2.06]	0.67 [0.28 - 1.61]

Abbreviations: NH=non-Hispanic; NE=not estimable

[a] Reference group: professional/management positions

[b] Reference group: professional/administrative/management industry

All models adjusted for age (18-30, 31-49, ≥50 years), gender (Women, Men), educational attainment (<high school, high school graduate, some college, ≥college), annual household income (<$35,000, $35,000-$74,999, $75,000+), region of residence (Northeast, Midwest, South, West), marital/co-habiting status (married/living with partner or cohabitating, divorced/widowed/separated, single/no live-in partner), health status (excellent/very good, good, fair/poor), alcohol consumption (never, former, current), and "ideal" cardiovascular health (never smoking/quit >12 months prior to interview, BMI 18.5-<25 kg/m, meeting physical activity guidelines, and no prior diagnosis of dyslipidemia, hypertension, or diabetes/prediabetes).

All estimates are weighted for the survey's complex sampling design. Boldface indicates statistically significant results at the 0.05 level

Overall models adjusted for race/ethnicity (NH-White, NH-Black, Hispanic/Latinx, and Asian).

*Estimates with particularly wide confidence intervals

Table S4. Adjusted Prevalence Ratios (95% Confidence Intervals) for the Association Between Occupational Class (compared to Professional/Management positions) and Industry of Employment (compared to the Professional/Administrative/Management Industry) and Serious Psychological Distress, Stratified by Race/Ethnicity and Annual Household Income, National Health Interview Survey, 2004-2018, (N=226,858) [a].

	Race/Ethnicity							
	Asian		NH-Black		Hispanic/Latinx		NH-White	
**Annual Household Income**	<$75,000 (N=6,796)	≥$75,000 (N=5,573)	<$75,000 (N=23,767)	≥$75,000 (N=5,948)	<$75,000 (N=32,607)	≥$75,000 (N=7,643)	<$75,000 (N=83,695)	≥$75,000 (N=60,829)
**Occupational Class** [b]								
Support services	1.26 [0.63 - 2.52]	1.75 [0.69 - 4.40]	**1.83 [1.15 - 2.92]**	0.48 [0.22 - 1.04]	1.22 [0.85 - 1.75]	1.05 [0.53 - 2.12]	1.12 [0.92 - 1.35]	1.28 [0.96 - 1.71]
Laborers	1.70 [0.74 - 3.91]	0.36 [0.08 - 1.59]	**1.74 [1.06 - 2.85]**	0.65 [0.27 - 1.58]	1.14 [0.78 - 1.68]	1.40 [0.68 - 2.88]	1.20 [0.98 - 1.47]	1.15 [0.79 - 1.69]
**Industry of Employment** [c]								
Manufacturing/Construction	0.78 [0.31 - 1.95]	1.57 [0.43 - 5.66]	0.76 [0.47 - 1.21]	0.52 [0.19 - 1.46]	0.76 [0.54 - 1.07]	0.82 [0.27 - 2.53]	**0.81 [0.66 - 0.99]**	0.79 [0.53 - 1.20]
Retail Trade	1.76 [0.70 - 4.48]	3.13 [0.80 - 12.3]	1.07 [0.64 - 1.79]	0.29 [0.06 - 1.39]	0.85 [0.57 - 1.25]	1.13 [0.33 - 3.92]	0.90 [0.72 - 1.11]	0.91 [0.58 - 1.45]
Finance/Information/Real Estate	0.64 [0.21 - 2.02]	1.81 [0.55 - 5.95]	0.55 [0.30 - 1.01]	0.31 [0.09 - 1.09]	0.75 [0.46 - 1.23]	1.58 [0.45 - 5.54]	**0.69 [0.53 - 0.90]**	0.67 [0.40 - 1.13]
Educational Services	0.89 [0.31 - 2.51]	2.57 [0.40 - 16.6]	0.83 [0.48 - 1.44]	0.17 [0.02 - 1.60]	0.65 [0.38 - 1.12]	1.49 [0.47 - 4.75]	**0.64 [0.49 - 0.83]**	1.12 [0.70 - 1.79]
Healthcare/Social Assistance	0.72 [0.27 - 1.87]	**4.35 [1.27 - 14.9]**	0.88 [0.56 - 1.40]	**0.18 [0.04 - 0.74]**	1.00 [0.70 - 1.44]	1.45 [0.49 - 4.36]	0.90 [0.73 - 1.11]	0.74 [0.46 - 1.17]
Accommodation/Food services	**0.30 [0.10 - 0.91]**	0.46 [0.05 - 4.37]	1.02 [0.60 - 1.73]	NE	0.98 [0.69 - 1.40]	0.41 [0.08 – 2.00]	1.01 [0.80 - 1.28]	1.26 [0.70 - 2.24]
Public Administration/Arts/Other services	1.23 [0.48 - 3.17]	1.19 [0.29 - 4.98]	0.61 [0.37 - 1.00]	**0.34 [0.12 - 0.99]**	**0.64 [0.42 - 0.96]**	1.65 [0.50 - 5.47]	0.82 [0.67 - 1.02]	0.71 [0.46 - 1.11]
Professional/Administrative/ Management								
Support services	NE	NE	1.86 [0.61 - 5.71]	NE	1.18 [0.54 - 2.55]	NE	**1.63 [1.07 - 2.49]**	1.27 [0.62 - 2.62]
Laborers	NE	NE	1.65 [0.54 - 5.04]	NE	0.92 [0.41 - 2.07]	NE	**1.94 [1.25 - 3.01]**	0.86 [0.30 - 2.52]
Manufacturing/Construction								
Support services	NE	NE	0.60 [0.19 - 1.92]	1.95 [0.54 - 7.07]	2.04 [0.87 - 4.80]	NE	0.89 [0.56 - 1.41]	1.75 [0.89 - 3.45]
Laborers	NE	NE	0.85 [0.29 - 2.51]	0.81 [0.15 - 4.37]	1.47 [0.68 - 3.18]	NE	1.15 [0.74 - 1.78]	1.22 [0.72 - 2.09]
Retail Trade								
Support services	NE	NE	1.07 [0.20 - 5.81]	NE	0.44 [0.13 - 1.54]	NE	1.66 [0.77 - 3.58]	0.98 [0.34 - 2.87]
Laborers	NE	NE	0.93 [0.14 - 6.08]	NE	0.46 [0.13 - 1.72]	NE	1.63 [0.68 - 3.89]	0.87 [0.21 - 3.58]
Finance/Information/Real Estate								

Support services	NE	NE	NE	NE	2.08 [0.71 - 6.12]	NE	0.78 [0.47 - 1.29]	1.00 [0.45 - 2.22]
Laborers	NE	NE	NE	NE	1.96 [0.58 - 6.55]	NE	0.55 [0.24 - 1.29]	1.75 [0.55 - 5.54]
Educational Services								
Support services	NE	NE	7.90 [0.99 - 63.1]	NE	0.28 [0.08 - 1.02]	NE	0.82 [0.41 - 1.67]	1.70 [0.58 - 4.96]
Laborers	NE	NE	5.81 [0.59 - 57.4]	NE	**0.09 [0.02 - 0.36]**	NE	1.33 [0.60 - 2.92]	0.43 [0.08 - 2.49]
Health Care/Social Assistance								
Support services	NE	NE	3.97 [0.54 - 29.1]	NE	**3.35 [1.05 - 10.7]**	NE	1.16 [0.57 - 2.38]	**3.86 [1.21 - 12.3]**
Laborers	NE	NE	3.55 [0.46 - 27.5]	NE	1.26 [0.27 - 5.79]	NE	1.09 [0.47 - 2.49]	4.32 [0.79 - 23.6]
Accommodation/Food Services								
Support services	NE	NE	3.19 [0.62 - 16.3]	NE	0.70 [0.24 - 2.08]	NE	1.42 [0.72 - 2.80]	1.65 [0.27 - 10.0]
Laborers	NE	NE	3.52 [0.82 - 15.1]	NE	1.10 [0.48 - 2.51]	NE	1.18 [0.65 - 2.12]	2.65 [0.52 - 13.6]
Support services	2.82 [0.47 - 16.9]	NE	2.39 [0.74 - 7.70]	NE	1.42 [0.46 - 4.44]	NE	1.31 [0.75 - 2.27]	1.38 [0.68 - 2.81]
Laborers	**10.12 [1.20 - 85.4]**	NE	1.24 [0.34 - 4.56]	NE	2.09 [0.61 - 7.20]	NE	0.94 [0.49 - 1.81]	1.29 [0.47 - 3.55]

[a] 18180 participants excluded for missing income data
[b] Reference group: professional/management positions
[c] Reference group: professional/administrative/management industry
Abbreviations: NH=non-Hispanic; NE=not estimable
All models adjusted for age (18-30, 31-49, ≥50 years), gender (Women, Men), educational attainment (<high school, high school graduate, some college, ≥college), annual household income (<$35,000, $35,000-$74,999, $75,000+), employment status (unemployed, employed), region of residence (Northeast, Midwest, South, West), marital/co-habiting status (married/living with partner or cohabitating, divorced/widowed/separated, single/no live-in partner), health status (excellent/very good, good, fair/poor), alcohol consumption (never, former, current), and "ideal" cardiovascular health (never smoking/quit >12 months prior to interview, BMI 18.5-<25 kg/m, meeting physical activity guidelines, and no prior diagnosis of dyslipidemia, hypertension, or diabetes/prediabetes).
All estimates are weighted for the survey's complex sampling design. Boldface indicates statistically significant results at the 0.05 level
Overall models adjusted for race/ethnicity (NH-White, NH-Black, Hispanic/Latinx, and Asian).

Table S5. Adjusted Prevalence Ratios (95% Confidence Intervals) for the Association Between Occupational Class (compared to Professional/Management positions) and Industry of Employment (compared to the Professional/Administrative/Management Industry) and Serious Psychological Distress, Stratified by Race/Ethnicity and Self-Reported Health Status, National Health Interview Survey, 2004-2018, (N=245,038).

	**Race/Ethnicity**							
	**Asian**		**NH-Black**		**Hispanic/Latinx**		**NH-White**	
	Excellent/Very Good/Good (N=12,857)	Fair/Poor (N=628)	Excellent/Very Good/Good (N=29,156)	Fair/Poor (N=3,002)	Excellent/Very Good/Good (N=39,535)	Fair/Poor (N=3,721)	Excellent/Very Good/Good (N=147,816)	Fair/Poor (N=8,269)
**Occupational Class [a]**								
Support services	1.61 [0.83-3.15]	1.11 [0.27-4.58]	1.09 [0.73-1.65]	2.28 [0.93-5.57]	1.19 [0.83-1.72]	1.22 [0.62-2.37]	1.12 [0.93-1.34]	1.36 [0.99-1.87]
Laborers	1.94 [0.97-3.90]	0.38 [0.07-2.04]	1.09 [0.68-1.72]	2.08 [0.81-5.31]	1.27 [0.86-1.87]	1.08 [0.53-2.22]	1.22 [0.99-1.51]	1.27 [0.92-1.75]
**Industry of Employment [b]**								
Manufacturing/Construction	1.16 [0.54-2.48]	0.88 [0.12-6.32]	0.65 [0.38-1.10]	1.00 [0.46-2.16]	0.78 [0.52-1.16]	0.85 [0.50-1.45]	0.81 [0.65-1.01]	0.88 [0.63-1.24]
Retail Trade	**3.43 [1.43-8.24]**	0.84 [0.11-6.37]	0.92 [0.52-1.63]	0.79 [0.37-1.72]	0.69 [0.44-1.10]	1.72 [0.88-3.37]	0.94 [0.74-1.19]	0.85 [0.58-1.23]
Finance/Information/Real Estate	1.22 [0.51-2.95]	0.59 [0.05-6.82]	**0.42 [0.21-0.83]**	0.80 [0.31-2.09]	0.80 [0.43-1.50]	1.36 [0.64-2.92]	**0.61 [0.46-0.82]**	0.93 [0.58-1.49]
Educational Services	1.82 [0.63-5.27]	0.38 [0.03-4.49]	0.69 [0.36-1.33]	0.63 [0.26-1.50]	0.76 [0.42-1.37]	0.79 [0.34-1.83]	0.80 [0.61-1.04]	0.64 [0.41-1.01]
Healthcare/Social Assistance	1.55 [0.52-4.59]	2.70 [0.42-17.2]	0.69 [0.41-1.16]	0.86 [0.42-1.73]	1.06 [0.72-1.57]	1.07 [0.53-2.19]	**0.79 [0.63-0.99]**	1.11 [0.76-1.61]
Accommodation/Food services	0.41 [0.16-1.08]	0.38 [0.04-3.9]	0.63 [0.34-1.16]	1.23 [0.55-2.74]	0.97 [0.64-1.48]	0.96 [0.53-1.74]	1.02 [0.78-1.33]	1.04 [0.68-1.58]
Public Administration/Arts/Other services	1.46 [0.62-3.46]	0.92 [0.12-6.84]	**0.50 [0.29-0.87]**	0.65 [0.28-1.52]	0.70 [0.43-1.15]	1.00 [0.50-2.00]	**0.76 [0.60-0.96]**	0.89 [0.62-1.29]
Professional/Administrative/Management								
Support services	NE	NE	1.06 [0.32-3.50]	NE	1.18 [0.51-2.72]	0.77 [0.15-3.88]	1.43 [0.91-2.25]	1.76 [0.81-3.83]
Laborers	NE	NE	1.31 [0.41-4.18]	NE	1.14 [0.39-3.34]	0.80 [0.20-3.25]	1.58 [1.00-2.50]	1.81 [0.82-3.99]
Manufacturing/Construction								
Support services	0.38 [0.07-1.91]	NE	1.15 [0.42-3.10]	0.52 [0.06-4.59]	0.83 [0.34-1.99]	3.22 [0.57-18.4]	0.98 [0.60-1.58]	2.11 [0.97-4.56]
Laborers	0.95 [0.27-3.36]	NE	1.29 [0.45-3.70]	0.35 [0.05-2.69]	0.78 [0.40-1.52]	1.73 [0.35-8.54]	1.13 [0.76-1.68]	1.79 [0.99-3.26]
Retail Trade								
Support services	NE	NE	1.03 [0.19-5.51]	NE	1.52 [0.18-12.9]	0.29 [0.05-1.58]	1.61 [0.74-3.50]	0.80 [0.23-2.84]
Laborers	NE	NE	1.21 [0.20-7.22]	NE	2.58 [0.29-22.9]	0.44 [0.06-2.95]	1.53 [0.63-3.71]	0.93 [0.22-4.00]

Finance/Information/Real Estate								
Support services	NE	NE	NE	NE	1.86 [0.66-5.28]	NE	0.93 [0.55-1.55]	0.64 [0.31-1.35]
Laborers	NE	NE	NE	NE	2.26 [0.61-8.43]	NE	0.73 [0.27-1.99]	0.81 [0.26-2.53]
Educational Services								
Support services	NE	NE	6.34 [0.78-51.8]	NE	0.60 [0.12-3.02]	NE	1.34 [0.66-2.72]	0.74 [0.22-2.52]
Laborers	NE	NE	6.70 [0.73-61.9]	NE	0.29 [0.05-1.83]	NE	1.01 [0.45-2.30]	1.44 [0.36-5.73]
Health Care/Social Assistance								
Support services	NE	NE	1.68 [0.41-6.84]	NE	2.03 [0.69-5.94]	NE	1.20 [0.60-2.42]	**3.86 [1.16-12.8]**
Laborers	NE	NE	1.39 [0.30-6.48]	NE	1.14 [0.27-4.83]	NE	1.44 [0.62-3.32]	2.47 [0.59-10.3]
Accommodation/Food Services								
Support services	NE	NE	1.45 [0.30-6.92]	NE	0.71 [0.17-2.89]	0.41 [0.03-5.31]	1.42 [0.71-2.82]	2.73 [0.56-13.4]
Laborers	NE	NE	2.12 [0.53-8.47]	NE	1.32 [0.47-3.69]	0.46 [0.04-4.98]	1.28 [0.70-2.33]	3.07 [0.66-14.2]
Public Administration/Arts/Other Services								
Support services	0.97 [0.32-2.92]	NE	1.30 [0.49-3.49]	NE	1.10 [0.27-4.52]	0.59 [0.05-6.57]	1.26 [0.77-2.07]	1.16 [0.46-2.92]
Laborers	2.72 [0.65-11.3]	NE	0.74 [0.21-2.60]	NE	1.65 [0.38-7.18]	0.84 [0.07-9.98]	1.30 [0.74-2.27]	0.43 [0.14-1.28]

[a] Reference group: professional/management positions
[b] Reference group: professional/administrative/management industry
Abbreviations: NH=non-Hispanic; NE=not estimable
All models adjusted for age (18-30, 31-49, ≥50 years), gender (Women, Men), educational attainment (<high school, high school graduate, some college, ≥college), annual household income (<$35,000, $35,000-$74,999, $75,000+), region of residence (Northeast, Midwest, South, West), marital/co-habiting status (married/living with partner or cohabitating, divorced/widowed/separated, single/no live-in partner), health status (excellent/very good, good, fair/poor), alcohol consumption (never, former, current), and “ideal” cardiovascular health (never smoking/quit >12 months prior to interview, BMI 18.5-<25 kg/m, meeting physical activity guidelines, and no prior diagnosis of dyslipidemia, hypertension, or diabetes/prediabetes).
All estimates are weighted for the survey’s complex sampling design. Boldface indicates statistically significant results at the 0.05 level
Overall models adjusted for race/ethnicity (NH-White, NH-Black, Hispanic/Latinx, and Asian).
